# Microbial Diversity and Characteristics of Kombucha as Revealed by Metagenomic and Physicochemical Analysis

**DOI:** 10.3390/nu13124446

**Published:** 2021-12-13

**Authors:** Mayank Kaashyap, Marc Cohen, Nitin Mantri

**Affiliations:** 1 The Pangenomics Group, School of Science, RMIT University, Melbourne, VIC 3083, Australia; mayank.kaashyap@rmit.edu.au; 2 The Good Brew Co., Brunswick, Melbourne, VIC 3056, Australia; info@drmarc.co; 3 The UWA Institute of Agriculture, The University of Western Australia, Perth, WA 6009, Australia

**Keywords:** bacteria, kombucha, metagenomics, microbial diversity, yeast, physiochemical

## Abstract

Kombucha is a fermented tea made from a Symbiotic Culture of Bacteria and Yeast (SCOBY) with a long history of use as a health tonic. It is likely that most health benefits come from the tea and fermentation metabolites from specific microbial communities. Despite its growing importance as a functional health drink, the microbial ecosystem present in kombucha has not been fully documented. To characterize the microbial composition and biochemical properties of ‘The Good Brew’ original base kombucha, we used metagenomics amplicon (16S rRNA and ITS) sequencing to identify the microbial communities at the taxonomic level. We identified 34 genera with 200 microbial species yet described in kombucha. The dominance of organic acid producing microorganisms *Acetobacter, Komagataeibacter* and *Starmerella* are healthy for the human gut and their glucose metabolising activities have a putative role in preventing conditions such as diabetes and obesity. Kombucha contains high protein (3.31 µg/mL), high phenolic content (290.4 mg/100 mL) and low sugars (glucose: 1.87 g/L; sucrose 1.11 g/L; fructose: 0.05 g/L) as compared to green tea. The broad microbial diversity with proven health benefits for the human gut suggests kombucha is a powerful probiotic. These findings are important to improve the commercial value of kombucha and uncover the immense prospects for health benefits.

## 1. Introduction

Kombucha has a long history throughout Asia and Europe as a fermented, naturally effervescent, drink that is reported to have multiple health enhancing effects. More recently in vitro and in vivo evidence suggest kombucha has health benefits that include, anti-microbial, antioxidant, detoxification, anti-tumour and immune-enhancing effects, along with enhancing gastrointestinal, hepatic cardiac and neurological function [1,2,3,4]. The microbial communities involved in the fermentation process produce a number of metabolites which make this tea beneficial to the human gut [5].

Traditionally kombucha is fermented in the presence of indigenous microorganisms in aerobic conditions and the resulting product is rich in organic acids (acetic, lactic acid, gluconic acid and glucuronic acids) and CO_2_ [6,7]. The process of kombucha fermentation is performed by a symbiotic colony of bacteria and yeast with the yeast anaerobically metabolising sucrose to produce simple sugars, alcohol and bacteria then aerobically metabolising these into organic acids and carbon dioxide [8]. Two important bacterial species include *Acetobacter*, which produces acetic acid and *Gluconobacter,* which produces gluconic acid.

Thus far, only culture-based or low-throughput methods have been used to dissect the microbial communities of kombucha; however, these studies used low volume fermentation and have several limitations which do not represent industrial scale production [7,9]. Kombucha has numerous health benefits that are likely to arise from various microbes and products of microbial activity; however, specific genera responsible for these activities are unclear. Recent advancements in Next-Generation Sequencing (NGS) technologies present an opportunity to classify bacterial and yeast species at an unprecedented high resolution and these technologies were used to analyse commercially produced kombucha fermented in Australia. This study is the most comprehensive microbial profiling of kombucha to date and presents findings that aide in understanding kombucha’s many health benefits.

## 2. Materials and Methods

### 2.1. Sample Collection

Samples were obtained directly from The Good Brew Kombucha Company, which produces kombucha from 75% green tea and 25% oolong tea. The kombucha was supplied in 330 mL glass bottles and kept refrigerated at 4 °C, which is how they are sold commercially.

### 2.2. Physiochemical Analysis of Kombucha

#### 2.2.1. Standard Physicochemical Analyses

Triplicate measurements were taken from distinct batches for each of the kombucha base samples. Water content and total soluble solids was measured with a Refracto 30GS (Mettler Toledo, Melbourne, Australia) and converted accordingly using the Chetaway Table [10]. Electrical conductivity was measured on SevenCompact Conductivity Meter S230 (Mettler Toledo, Melbourne, Australia) at 20 °C in 20% (*w/v*) kombucha solution in Milli-Q water. Ash content was obtained by placing 5 mL of kombucha in a crucible (Labec, Sydney, Australia) and heating at 600 °C overnight in a muffle furnace. pH measurement of kombucha solution was performed using a pH meter. Visual colour was assessed following a method described by [11]. A chromameter CR400/410 (Konica Minolta, New South Wales, Australia) was used for CIE L∗, a∗, b∗ measurements of kombucha samples, where L∗: lightness, −a∗: greenness, a∗: redness and b∗: yellowness, as compared with the white tile background. Colour intensity of 50% (*v/v*) kombucha solution, filtered through 0.45 µm filter to remove any coarse particles, was measured as described by [12]. Spectrophotometric absorbance was taken at 450 nm using a Lambda 35 UV–vis spectrophotometer from Perkin Elmer (Waltham, MA, USA).

#### 2.2.2. Reducing Sugars

Amounts of D-glucose and D-fructose in kombucha were determined with Megazyme’s Assay Kit (K-SUFRG 06/14). D-glucose was determined by utilising hexokinase and glucose-6-phosphate without hydrolysing sucrose. D-fructose was determined after the determination of D-glucose following isomerisation with phosphoglucose isomerase. Samples was analysed in triplicate and the mean was expressed as mL/100 mL kombucha.

#### 2.2.3. Protein Content

Total protein content was determined with Thermo Scientific™ Coomassie (Bradford, UK) Protein Assay Kit. Twenty µL of 10% (*w*/*v*) kombucha solution was pipetted into a microplate. Then, 250 µL of Coomassie reagent was added and the plate was put in a shaker to incubate at ambient temperature for 10 min. Absorbance was measured at 595 nm with a Polar microplate reader, against a standard solution of bovine serum albumin (0–100 µg/mL). Milli-Q water was used as blank, and each sample was analysed in triplicate, with the mean being expressed in mL/100 mL kombucha.

#### 2.2.4. Total Phenolic Content

Total phenolic content was determined with the Folin-Ciocalteu method [13]. Kombucha sample (5 mL) was diluted to 50 mL with Milli-Q water and filtered through Whatman No. 1 paper. Solution (0.5 mL) was mixed with 2.5 mL of 0.2 N Folin-Ciocalteu reagent (Sigma-Aldrich, North Ryde, Australia) for 5 min and 2 mL of 75 g/L sodium carbonate was then added. Mixture was incubated at ambient temperature for 2 h before reading with a Lambda 35 UV–vis spectrophotometer. Absorbance was measured at 760 nm against an ethanol blank. Gallic acid was used to produce a standard curve from 0 to 100 mg/L. All analyses were carried out in triplicate and the mean was expressed in mg of gallic acid equivalents (GAE)/100 mL kombucha.

#### 2.2.5. Fourier Transform Infrared Spectroscopy

A spectrometer equipped with a MIRacleTM ZnSe single reflection ATR plate (Perkin-Elmer, Norwalk, CT, USA) was used to record FTIR spectra for kombucha. In doing so, 0.5 mL was placed onto the measuring plate and scanned forty times from 4000 to 650 cm^−1^ at a resolution of 4 cm^−1^ at ambient temperature.

#### 2.2.6. Modulated and Micro-Differential Scanning Calorimetry

Heat capacity measurements to determine the calorimetric glass transition temperature was conducted on Q2000 calorimeter (TA instruments, New Castle, DE, USA), with nitrogen purge gas at a flow rate of 50 mL/min. Ten mL of kombucha was loaded into a hermetic aluminium pan and equilibrated for 20 min at 20 °C. Samples were cooled to −90 °C and heated up to 30 °C at 1 °C/min. Triplicate measurements were performed at modulation amplitude of 0.53 °C every 40 s.

### 2.3. Microbial Analysis of Kombucha Samples

Metagenomics analysis of all kombucha sample with three replicates was performed to identify microbial type and count in each sample. High quality good yield DNA was extracted using PureLink Microbiome DNA Purification Kit (ThermoFisher Scientific, Waltham, MA, USA) from the kombucha samples and used as template for 16S and ITS metagenomic analysis using the Illumina MiSeq platform (Illumina, San Diego, CA, USA). Library preparation was performed using the V3–V4 hypervariable region of the 16S rRNA gene amplified with primers 341F (CCTACGGGRSGCAGCAG) and 806R (GGACTACHVGGGTWTCTAAT). ITS library was made by amplifying the targets ITS1 (CTTGGTCATTTAGAGGAAGTAA) and ITS2 (GCTGCGTTCTTCATCGATGC). The PCR reaction was performed using Platinum Taq Polymerase (Invitrogen, USA) with the following conditions: 95 °C for 5 min, 25 cycles of 95 °C for 45 s, 55 °C for 30 s and 72 °C for 45 s and a final extension of 72 °C for 2 min. Library preparation (attachment of TruSeq adapters, purification with AMPureXP beads and qPCR quantification) was performed using Illumina 16S Library Preparation Protocol (Illumina Technical Note 15044223 Rev. B). Sequencing was performed using MiSeq Reagent Kit v3 with 2 × 300 bp paired-end reactions.

Sequencing data for each sample were processed. Initially, the sequencing output was analysed by a read quality filter, which removes reads with an average Phred score <20 followed by a clustering of 100% identical reads. To remove putative chimeric sequences, clusters with less than 5 reads were excluded from further analysis. The remaining good-quality sequences were further clustered at 97% similarity to assign taxonomic classification using BaseSpace 16S metagenomics app (Illumina, San Diego, CA, USA). Classification of the reads was performed using a high-performance version of the (RDP) naive Bayesian taxonomic classification algorithm [14]. Taxonomic classification was made by comparing them with a custom Ref Seq 16S v3 rRNA DADA2 Database and UNITE Fungal ITS Database v7.2. Sequences were taxonomically assigned with at least 99% identity in the reference database. To evaluate the microbial community shifts among samples, the relative abundance of each genus was compared across the samples.

## 3. Results

### 3.1. Metagenomics Sequencing and Assembly

Each sample was deep sequenced to 10 million high quality reads and 100% reads passed the quality check. Out of these, 99.98% reads were mapped to the RefSeq RDP 16S v3 DADA2 Database (Table 1). High percentage of reads classified to bacterial taxonomic levels from kingdom (99.98%) to species (52.82%). A greater number of reads assembled as operational taxonomic units; the dominant genera have 100,000 to 700 hits while dominant species show 65,000 to 500 hits to the dataset.

#### 3.1.1. Dissecting Bacterial Species in Kombucha Using 16S Metagenomics Analysis

**Figure 1:**
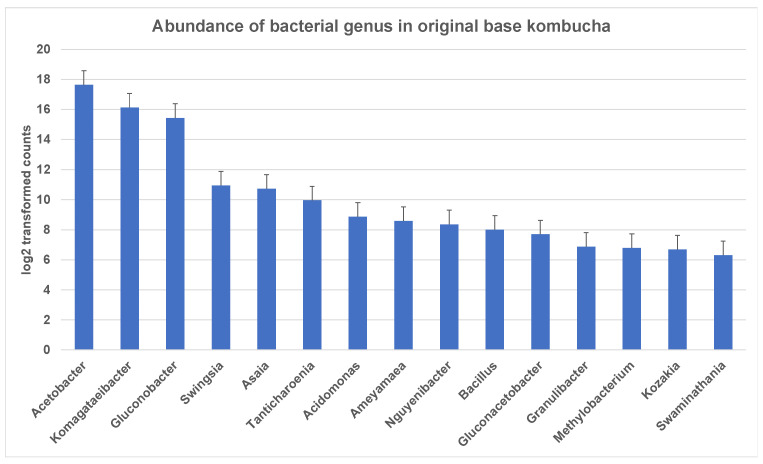
Most abundant bacterial genus in original base kombucha. *Acetobacter* being the most dominant genus followed by *Komagataeibacter* and *Gluconobacter*. The error bars are standard error (SE) calculated across the triplicate values of samples.

The entropy of species-level classifications in the sample based on Shannon Species Diversity measures was 1.38 for kombucha. A total of 198 bacterial genera were identified in ‘The Good Brew’ original base kombucha samples. These genera included 189 bacterial species across the sample replicates. Notably, most of these genera could not be identified at the species level. The most dominant genera in original base kombucha are *Acetobacter* (57% reads classified to genus), *Komagataeibacter* (34% reads classified to genus), and *Gluconobacter* (7% reads classified to genus) (Figure 1). These belong largely to family Acetobacteraceae which had 99% of reads classified at this level.

**Figure 2:**
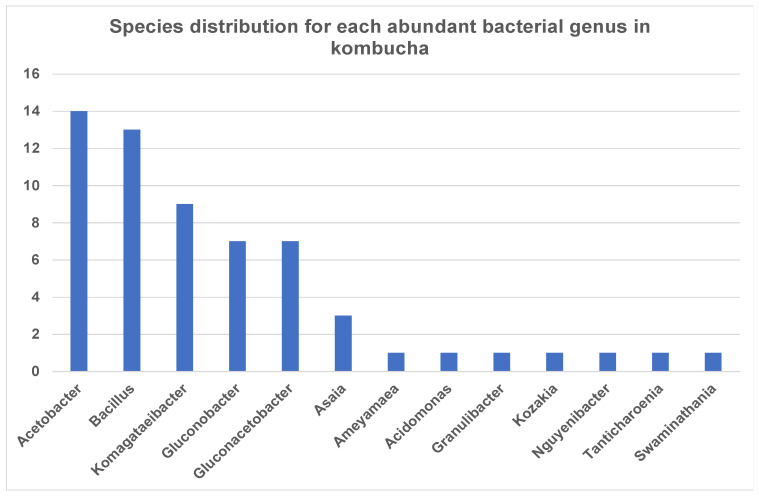
Number of bacterial species each abundant genus has in kombucha. *Acetobacter* genus has 14 different species; *Bacillus* genus has 13 different species; *Komagataeibacter* genus has 9 different species in kombucha samples.

Interestingly, the most dominant genus Acetobacter showed high species diversity (fourteen species) followed by *Bacillus* (thirteen species) and *Komagataeibacter* (nine species) (Figure 2). Other genera such as *Asaia* and *Kozakia* showed less species diversity and have only one species, but these are highly abundant across the samples.

**Figure 3:**
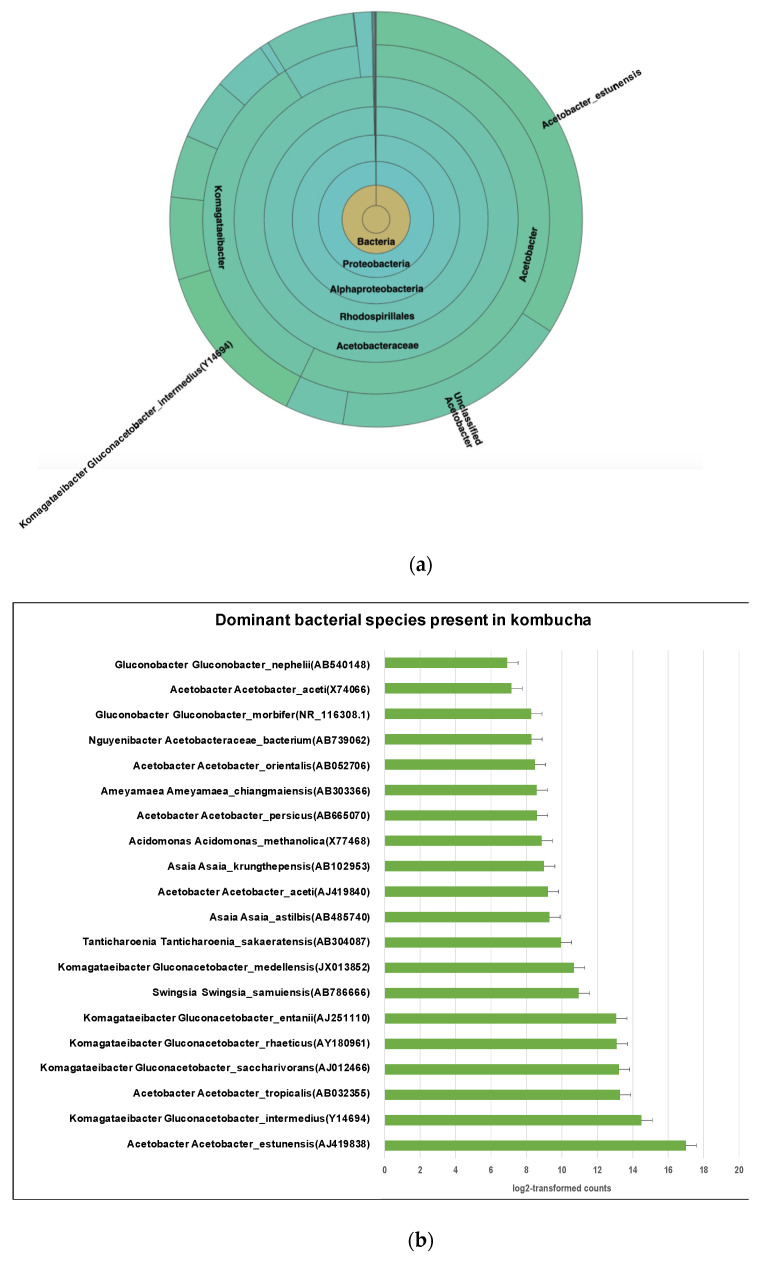
(**a**) Sunburst chart shows the relative abundance of the bacterial classification within each taxonomic level. Kingdom: Bacteria; Phylum: Proteobacter; Class: Alphaproteobacteria; Order: Rhodospirillales; Family: Acetobacteraceae; Genus: *Acetobacter* and *Komagataeibacter*; Species *Acetobacter*_*estunensis* and *Komagataeibacter Gluconacetobacter_intermedius*. High- percent of reads (57.37%) match with dominant genus *Acetobacter* while 34.16% reads are assigned to genus *Komagataeibacter.* (**b**) Different dominant bacterial species in kombucha samples. *Acetobacter Acetobacter estunensis* is the most abundant followed by *Komagataeibacter Gluconacetobacter_intermedius* species. The error bars are standard error (SE) calculated across the triplicate values of samples.

The most abundant species is Acetobacter *Acetobacter estunensis* (AJ419838) followed by *Komagataeibacter Gluconacetobacter*_*intermedius* (Y14694) (Figure 3a). A total 50.83% reads classified to species Acetobacter estunensis, 19% of reads classified to *Komagataeibacter Gluconacetobacter*_intermedius, 7% reads classified to *Komagataeibacter Gluconacetobacter*_entanii, 7% reads classified to *Komagataeibacter* Gluconacetobacter_*saccharivorans*, 6% reads classified to Acetobacter *Acetobacter*_*tropicalis*, and 6% reads classified to *Komagataeibacter Gluconacetobacter*_*rhaeticus* (Figure 3b). This suggests the kombucha samples were dominated by Acetic Acid Bacteria (AAB) species. These bacteria have essential roles in oxidising sugar and alcohol to produce acetic acid during the kombucha fermentation.

#### 3.1.2. Dissecting Yeast Species in Kombucha Using ITS (Internal Transcribed Spacer) Metagenomics Analysis

**Figure 4:**
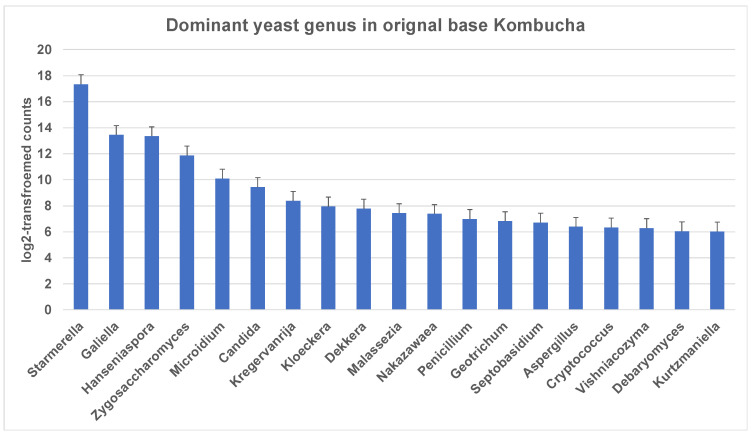
Most dominant yeast genera across the kombucha samples. More reads match to the genus *Starmerella* followed by *Galiella* and *Hanseniaspora*. The error bars are standard error (SE) calculated across the triplicate values of samples.

**Figure 5:**
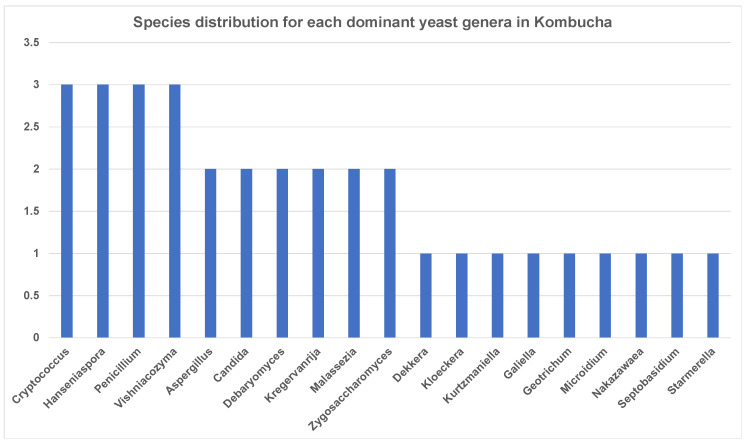
Abundance of yeast species each dominant genus has in kombucha. On average, genus *Cryptococcus*, *Hanseniaspora*, *Penicillium* and *Vishniacozyma* show three different species across kombucha samples.

**Figure 6:**
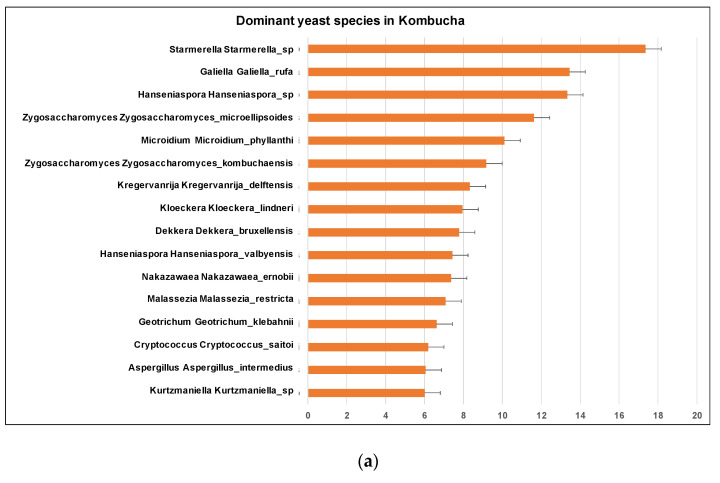

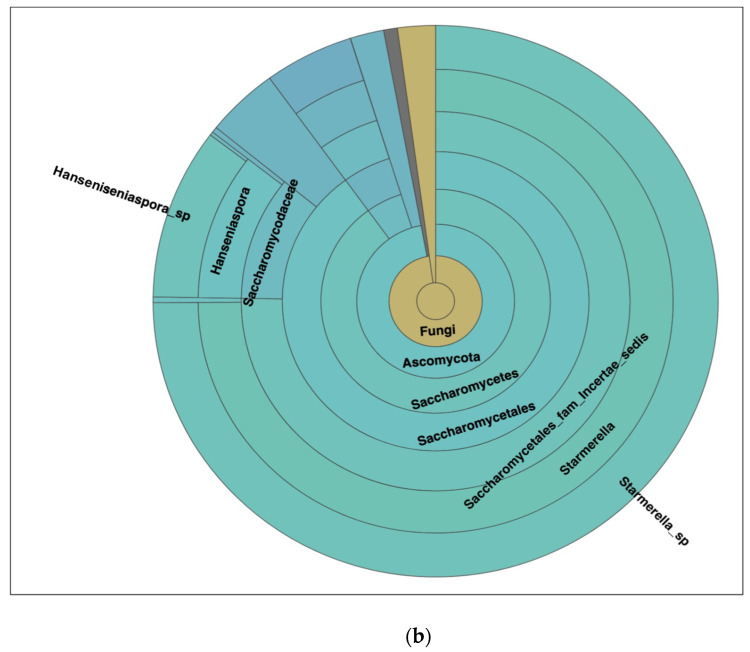
(**a**) Different dominant yeast species in kombucha samples. *Starmerella Starmerella* is the most abundant species followed by *Galiella Galiella rufa* species. The error bars are standard error (SE) calculated across the triplicate values of samples. (**b**) Sunburst chart shows the relative abundance of the yeast classification within each taxonomic level. Kingdom: Fungi; Phylum: Ascomycota; Class: Saccharomycetes; Order:Sccharomycetales; Family: Saccharomycetales_fam_Incertae_sedis; Genus: *Starmerella* and *Hanseniaspora*; Species *Starmerella_sp* and *Hanseniaspora sp*.

Similar to 16S results, a greater number of ITS-metagenomic reads (97%) were classified to the taxonomic level using UNITE fungal ITS database v 7.2. A total of 117 yeast genera were identified which comprised 139 yeast species. The most dominant genera found in original base kombucha are *Starmerella*, *Galiella*, and *Hanseniaspora* (Figure 4). Total 77% reads classified to genus *Starmerella*, 10% to *Hanseniaspora*, 5% to *Galiella* and *3% to Zygosaccharomyces* genus. These genera showed high species diversity where genus *Hanseniaspora* had three species, *Zygosaccharomyces* had two and *Starmerella* had only one species (Figure 5). *Starmerella* sp. has been reported in wine fermentation and it significantly contributes to the microbial ecology of the fermentation. These yeast species cope well with sugar niches and are reported to produce low amounts of acetic acid, glycerol and acetaldehydes as a result of sugar fermentation. The most dominant yeast species is *Starmerella* sp. with 78% ITS reads classified to this species. The second most dominant species is *Galiellea rufa* (10% reads), *Hanseniaspora* sp. (6% reads) and *Zygosaccharomyces microellipsoides* (3% reads) classified to these species (Figure 6a,b).

### 3.2. Physiochemical Properties of Kombucha

The pH of the kombucha samples was highly acidic (pH: 3.00) and reddish yellow in colour (L∗: lightness 92.98, a∗: redness 0.10; b∗: yellowness: 3.64). These samples had an electrical conductivity of 669 µS/cm and 2.9 Brix% of refractive index. An amount of 1% Brix equates to 1 gm of sucrose in 100 mL of sample. The total soluble measured as absorbance at 450 nm and ash content was 0.23 and 2.26 g/10 mL, respectively. Importantly, a high refractive index value suggests a high amount of solutes in samples and it can be concluded that samples contain various sugars (Table 2).

#### 3.2.1. Sugar Estimate

Sugars are an important substrate for the microbial species to ferment the important metabolites in kombucha. These essentially comprise of disaccharide (sucrose) and monosaccharides (fructose and glucose). The sugar content was estimated using enzymatic hydrolysis of sucrose read as absorbance at 340 nm. Kombucha samples had sucrose content (1.11 g/L), glucose content (1.87 g/L) and fructose content (0.05 g/L).

#### 3.2.2. Protein Content

Protein content is a measure of total amino acids and enzymes secreted during the fermentation. The protein content of kombucha samples was 3.31 µg/mL.

#### 3.2.3. Phenolic Content

Phenolic content is a measure of antioxidant activity. The total phenolics estimated in kombucha samples was 290 mg/mL. It has been observed that high colour values indicate high phenolic content, and these values correlate well with the colour values of kombucha.

#### 3.2.4. Colony Forming Unit (CFU/mL)

**Figure 7:**
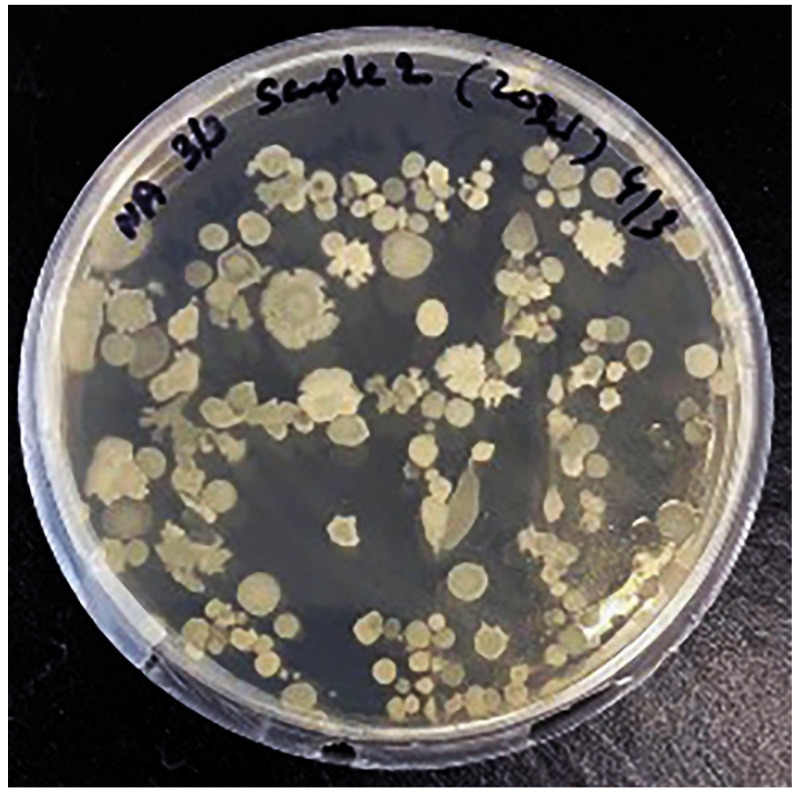
Colony Forming Unit (CFU/200 µL) of kombucha samples.

Kombucha samples were recorded for absorbance at 600 nm to estimate the colony forming units (CFU) per mL. Calculated volume was plated on nutrient agar to observe the microbial growth. Total CFU observed were 108 per mL. Interestingly, more yeast colonies were observed to grow in kombucha samples (Figure 7).

#### 3.2.5. Fourier Transform Infrared Spectroscopy (FTIR) Analysis

**Figure 8:**
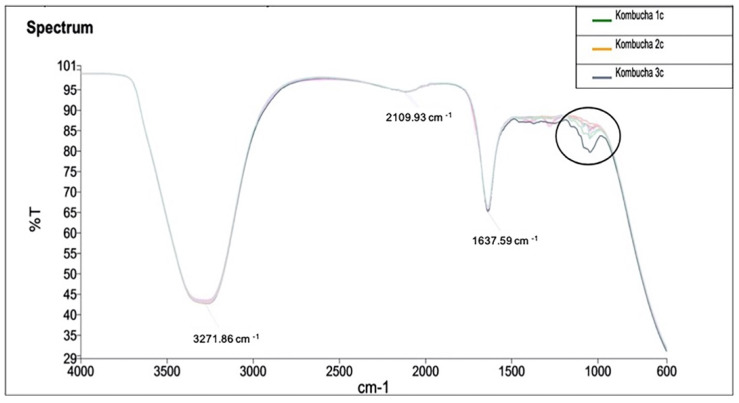
FTIR analysis of kombucha samples to identify the vibrations and absorbance of chemical functional groups. The spectrum is recorded at wavelength range of 4000 to 600 cm^−1^ by measuring the infrared transmitted (%T). A differential peak is observed at wavelength between 1100 cm^− 1^ to 1000 cm^−1^.

Infrared spectrum of the samples and identified peaks at 1000 cm^−1^ indicate the variance in —OH functional group across the samples (Figure 8). Samples have shown a differential peak at 1100 cm^−1^ to 1000 cm^−1^. This peak corresponds to group C–O, and therefore, the samples vary in a compound class of primary and secondary alcohol and aromatic ester groups.

#### 3.2.6. Differential Scanning Colorimeter (DSCQ2000) Glass-Transition Analysis

**Figure 9:**
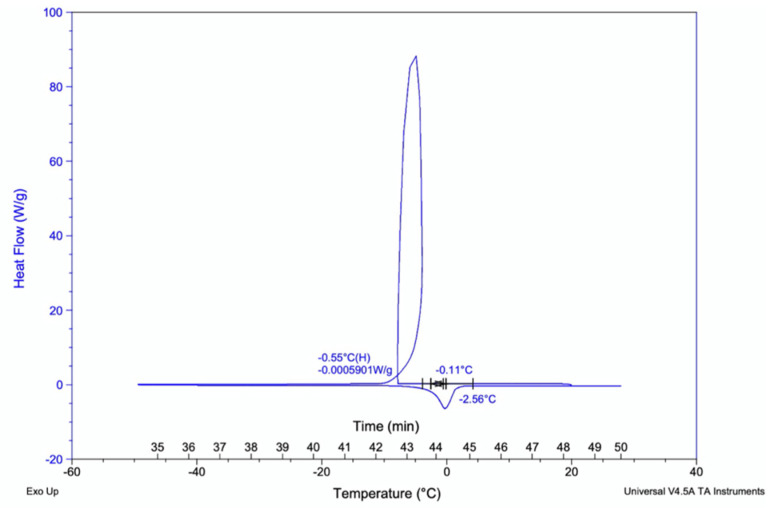
Glass-transition temperature curve for kombucha samples. The polymers begin transition to glass state at temperatures −2.56 °C to −0.11 °C. Heat flow (W/g).

Importantly, the glass transition temperature of kombucha samples were recorded to find the temperature curves of polymers present. This is the temperature at which polymers begin to change form denoted as Tg temperature. The sample replicates showed similar temperature curves between −2.56 °C to −0.11 °C for 2.0 mg of liquid samples (Figure 9).

## 4. Discussion

Kombucha has potential benefits to human health due to its rich probiotic characteristics. A comprehensive 16S metagenomics analysis conducted on The Good Brew kombucha base samples revealed a total of 200 different species including dominant 20 bacterial species and 16 yeast species were identified. Amongst these, *Acetobacter*, *Bacillus*, *Starmerella* and *Komagataeibacter* and *Gluconobacter* are the most prominent. These species form symbiosis with beneficial strains in the human gut and can inhibit the growth of infectious microorganisms [1,15]. Acetobacter produce by-products such as acetic and gluconic acid which are effective at inhibiting the growth of some common pathogenic bacteria that can cause the stomach upsets, sickness and diarrhoea associated with food poisoning. Importantly, these properties are attributes of probiotics with proven health benefits, which include a nonspecific mechanism to inhibit the growth of pathogenic bacteria in human gastrointestinal tract [16,17,18,19]. Further, gluconic acids produced by *Komagataeibacter* have important function as mineral supplements to treat hypocalcaemia, hypomagnesaemia and anaemia [20,21]. The high glucose conversion efficiency and survival rate in acidic pH suggests kombucha may play a role in preventing diabetes and obesity [22,23]. The species identified in this study and their crosstalk with other dominant yeast and bacterial species qualify as probiotics that have not been previously described. These findings are also supported by studies on kombucha and apple vinegar that shows *Komagataeibacter xylinus* is the main microorganism in them and has qualifying properties as a probiotic [23,24].

Commercial probiotics include seven genera of live and dead microorganisms such as *Lactobacillus*, *Bifidobacterium*, *Saccharomyces*, *Streptococcus*, *Enterococcus*, *Escherichia*, and *Bacillus.* One of the most important criteria for selecting a microorganism as a probiotic is its ability to stay alive within the gastric environment such as low oxygen pressure and the presence of bile salts and acid [25,26,27]. Importantly, we report 34 genera of live microorganisms in kombucha with the ability to thrive in the acidic environment of the human gut and establish a symbiotic association essential for health benefits [28,29,30].

Apart from resistance to gastrointestinal enzymes, an essential prerequisite of a probiotic is the ability to reduce cholesterol [31]. In addition to fermenting fructose faster than glucose or sucrose, *Starmerella* require lipid derivatives for growth and thus help lower cholesterol level [32,33,34]. The dominance of *Starmerella* and low quantity of fructose in our findings, suggests that kombucha could provide better health benefits over other probiotics in preventing conditions such as hypercholesteremia. Further, we identified 108 CFU/mL from live cultures of kombucha, which is 10-fold higher than reported in other kombucha studies, suggesting that these cells are viable and are sustained in human gut to provide health benefits [30].

The kombucha was produced from water, green tea, oolong tea, sugar and a starter culture of symbiotic bacteria and yeast. The fermentation process leads to the bioactivation of nutrients and phytochemicals and the production of additional compounds that significantly add to the health benefits of tea. Green tea has numerous health benefits due to its rich polyphenolic content. More recent in-vitro and in-vivo scientific studies have further supported the role polyphenols in modulating gut microbiota [35,36]. The rich polyphenols in green tea are of great interest for drug discovery and widely known for their hypocholesteremic, antibiotic, anticarcinogen and hypoglycaemic properties [37,38,39,40]. Approximately, 100 mL of green tea contains 125 mg of polyphenols [41,42]. Interestingly, we identified 290 mg/mL of total phenols in kombucha base samples which is 2.5-fold higher than green tea, which suggests the fermentation process produces additional polyphenols. Studies have indicated that phenolics are directly correlated with the antioxidant activity and phenolic content of fermented products is higher than unfermented products [43,44]. Although high in important phenolics for healthy gut, green tea phenols are relatively volatile, poorly absorbed in the intestine and readily converted to other metabolites by the gut microbiota and green tea is an unfermented drink with low microbial diversity [34]. Kombucha has a higher content of phenolic compounds, proteins and organic acids as well as broader microbial diversity than green tea and is therefore likely to be a more effective probiotic and bestow significant health benefits beyond those of green tea.

Interestingly, the esters detected in FTIR analysis indicate increased yeast-induced production of esters. Apart from imparting flavour and aroma to kombucha, these ester groups also have an important role as antibacterial food additives by inhibiting bacterial growth [1]. Esters are hydrophobic in nature which allows the electrostatic interactions with the bacterial cell components resulting in loss of cell viability [45]. This suggests an important crosstalk between the yeast and bacterial communities to enrich the healthy properties and shelf life of kombucha. It would be interesting to observe the differential peak of these aromatic groups between different kombucha samples varying in various herbal infusions, fermentation time and conditions. The comprehensive biochemical analysis presented in this study would be an important benchmark to identify the biochemical fingerprints of different kombuchas and should extend to other probiotic foods which have microbial profiles resulting from similar fermentation formulations.

Kombucha is increasingly gaining importance as a health drink and diet supplement, yet the health enhancing properties of kombucha remain undefined. This comprehensive study reports on more than 200 microbial species and the downstream physio-chemical analysis of kombucha and suggests that kombucha is a source of bioactive compounds, essential microbiota and important functional attributes that contribute to its role as a dietary supplement and natural probiotic.

## 5. Conclusions

Kombucha has broad microbial diversity enriched with organic acid producing microorganisms, laying the groundwork for immense potential for health benefits. The dominance of yeast and bacterial strains and their crosstalk are essential for bioavailability in providing benefits to the healthy human gut. Kombucha cultures have higher efficiency to metabolise sugars and produce essential metabolites as compared to other fermented health foods. Further comparative studies are needed to understand how variations in microbial diversity, brewing process, fermentation time, herbs and fruits in a secondary infusion affect the quality of kombucha and its impact on health.

## Figures and Tables

**Table 1:** Read mapping and classification statistics.

Taxonomic Level	Reads PF Classified to Taxonomic Level	% Reads PF Classified to Taxonomic Level
Kingdom	157,818	99.98%
Phylum	157,771	99.95%
Class	157,693	99.90%
Order	157,637	99.86%
Family	157,570	99.82%
Genus	153,947	97.52%
Species	83,375	52.82%

**Table 2:** Physiochemical properties of kombucha.

Kombucha BASE	Estimate
Protein (µg/mL)	3.31
Phenol (mg/100mL)	290.40
Glucose (g/L)	1.87
Fructose (g/L)	1.11
Sucrose (g/L)	0.053
Electrical Conductivity (µS/cm)	669.00
pH	3.00
Refractive Index (Brix%)	2.90
CFU/mL (650 nM)	4.9 × 10^7^
CFU/mL (live culture)	108.00
Total Solubles (A450 nm)	0.23
Ash Content (g/10mL)	2.26

## Data Availability

Data will be uploaded to NCBI GEO Database. Please contact corresponding author.

## References

[1] Al-Mohammadi A.R., Ismaiel A.A., Ibrahim R.A., Moustafa A.H., Abou Zeid A., Enan G. (2021). Chemical Constitution and Antimicrobial Activity of Kombucha Fermented Beverage. Molecules.

[2] Jayabalan R., Malbasa R.V., Loncar E.S., Vitas J.S., Sathishkumar M. (2014). A Review on Kombucha Tea-Microbiology, Composition, Fermentation, Beneficial Effects, Toxicity, and Tea Fungus. Compr. Rev. Food Sci. Food Saf..

[3] Kapp J.M., Sumner W. (2019). Kombucha: A systematic review of the empirical evidence of human health benefit. Ann. Epidemiol..

[4] Martinez-Leal J., Ponce-Garcia N., Escalante-Aburto A. (2020). Recent Evidence of the Beneficial Effects Associated with Glucuronic Acid Contained in Kombucha Beverages. Curr. Nutr. Rep..

[5] Chakravorty S., Bhattacharya S., Chatzinotas A., Chakraborty W., Bhattacharya D., Gachhui R. (2016). Kombucha tea fermentation: Microbial and biochemical dynamics. Int. J. Food Microbiol..

[6] Villarreal-Soto S.A., Bouajila J., Pace M., Leech J., Cotter P.D., Souchard J.P., Taillandier P., Beaufort S. (2020). Metabolome-microbiome signatures in the fermented beverage, Kombucha. Int. J. Food Microbiol..

[7] Coton M., Pawtowski A., Taminiau B., Burgaud G., Deniel F., Coulloumme-Labarthe L., Fall A., Daube G., Coton E. (2017). Unraveling microbial ecology of industrial-scale Kombucha fermentations by metabarcoding and culture-based methods. FEMS Microbiol. Ecol..

[8] May A., Narayanan S., Alcock J., Varsani A., Maley C., Aktipis A. (2019). Kombucha: A novel model system for cooperation and conflict in a complex multi-species microbial ecosystem. PeerJ.

[9] Reva O.N., Zaets I.E., Ovcharenko L.P., Kukharenko O.E., Shpylova S.P., Podolich O.V., de Vera J.P., Kozyrovska N.O. (2015). Metabarcoding of the kombucha microbial community grown in different microenvironments. AMB Express.

[10] Chataway H. (1935). Honey tables showing the relationship between various hydrometer scales and refractive index to moisture content and weight per gallon of honey. Can. Bee J..

[11] Bertoncelj J., Doberšek U., Jamnik M., Golob T. (2007). Evaluation of the phenolic content, antioxidant activity and colour of Slovenian honey. Food Chem..

[12] Beretta G., Granata P., Ferrero M., Orioli M., Maffei Facino R. (2005). Standardization of antioxidant properties of honey by a combination of spectrophotometric/fluorimetric assays and chemometrics. Anal. Chim. Acta.

[13] Singleton V.L., Orthofer R., Lamuela-Raventós R.M. (1999). [14] Analysis of total phenols and other oxidation substrates and antioxidants by means of folin-ciocalteu reagent. Methods in Enzymology.

[14] Wang Q., Garrity G.M., Tiedje J.M., Cole J.R. (2007). Naïve Bayesian Classifier for Rapid Assignment of rRNA Sequences into the New Bacterial Taxonomy. Appl. Environ. Microbiol..

[15] Vitas J., Malbasa R., Grahovac J., Loncar E. (2013). The antioxidant activity of kombucha fermented milk products with stinging nettle and winter savory. Chem. Ind. Chem. Eng. Q..

[16] Sanders M.E. (2015). Probiotics in 2015: Their Scope and Use. J. Clin. Gastroenterol..

[17] Hill C., Guarner F., Reid G., Gibson G.R., Merenstein D.J., Pot B., Morelli L., Canani R.B., Flint H.J., Salminen S. (2014). The International Scientific Association for Probiotics and Prebiotics consensus statement on the scope and appropriate use of the term probiotic. Nat. Rev. Gastroenterol. Hepatol..

[18] Gomes R.J., Borges M.d.F., Rosa M.d.F., Castro-Gómez R.J.H., Spinosa W.A. (2018). Acetic Acid Bacteria in the Food Industry: Systematics, Characteristics and Applications. Food Technol. Biotechnol..

[19] Zmora N., Zilberman-Schapira G., Suez J., Mor U., Dori-Bachash M., Bashiardes S., Kotler E., Zur M., Regev-Lehavi D., Brik R.B. (2018). Personalized Gut Mucosal Colonization Resistance to Empiric Probiotics Is Associated with Unique Host and Microbiome Features. Cell.

[20] Saichana N., Matsushita K., Adachi O., Frébort I., Frebortova J. (2015). Acetic acid bacteria: A group of bacteria with versatile biotechnological applications. Biotechnol. Adv..

[21] Cañete-Rodríguez A.M., Santos-Dueñas I.M., Jiménez-Hornero J.E., Ehrenreich A., Liebl W., García-García I. (2016). Gluconic acid: Properties, production methods and applications—An excellent opportunity for agro-industrial by-products and waste bio-valorization. Process. Biochem..

[22] Amarasinghe H., Weerakkody N.S., Waisundara V.Y. (2018). Evaluation of physicochemical properties and antioxidant activities of kombucha “Tea Fungus” during extended periods of fermentation. Food Sci. Nutr..

[23] Lavasani P.S., Motevaseli E., Sanikhani N.S., Modarressi M.H. (2019). Komagataeibacter xylinus as a novel probiotic candidate with high glucose conversion rate properties. Heliyon.

[24] Bellassoued K., Ghrab F., Makni-Ayadi F., Van Pelt J., Elfeki A., Ammar E. (2015). Protective effect of kombucha on rats fed a hypercholesterolemic diet is mediated by its antioxidant activity. Pharm. Biol..

[25] Ma C., Zhang S., Lu J., Zhang C., Pang X., Lv J. (2019). Screening for Cholesterol-Lowering Probiotics from Lactic Acid Bacteria Isolated from Corn Silage Based on Three Hypothesized Pathways. Int. J. Mol. Sci..

[26] Ding W., Shi C., Chen M., Zhou J., Long R., Guo X. (2017). Screening for lactic acid bacteria in traditional fermented Tibetan yak milk and evaluating their probiotic and cholesterol-lowering potentials in rats fed a high-cholesterol diet. J. Funct. Foods.

[27] Diguță C.F., Nițoi G.D., Matei F., Luță G., Cornea C.P. (2020). The Biotechnological Potential of *Pediococcus* spp. Isolated from Kombucha Microbial Consortium. Foods.

[28] Rajkowska K., Kunicka-Styczyńska A. (2010). Probiotic properties of yeasts isolated from chicken feces and kefirs. Pol. J. Microbiol..

[29] Rondanelli M., Faliva M.A., Perna S., Giacosa A., Peroni G., Castellazzi A.M. (2017). Using probiotics in clinical practice: Where are we now? A review of existing meta-analyses. Gut Microbes.

[30] Tu C., Hu W., Tang S., Meng L., Huang Z., Xu X., Xia X., Azi F., Dong M. (2020). Isolation and identification of Starmerella davenportii strain Do18 and its application in black tea beverage fermentation. Food Sci. Hum. Wellness.

[31] Şanlidere Aloğlu H., Demir Özer E., Öner Z. (2016). Assimilation of cholesterol and probiotic characterisation of yeast strains isolated from raw milk and fermented foods. Int. J. Dairy Technol..

[32] Petsiou E.I., Mitrou P.I., Raptis S.A., Dimitriadis G.D. (2014). Effect and mechanisms of action of vinegar on glucose metabolism, lipid profile, and body weight. Nutr. Rev..

[33] Mitrou P., Raptis A.E., Lambadiari V., Boutati E., Petsiou E., Spanoudi F., Papakonstantinou E., Maratou E., Economopoulos T., Dimitriadis G. (2010). Vinegar Decreases Postprandial Hyperglycemia in Patients With Type 1 Diabetes. Diabetes Care.

[34] Lozano J.d.D., Ju·rez-Flores B., Pinos-RodrÌguez J., Aguirre-Rivera J.R., ¡lvarez-Fuentes G. (2012). Supplementary effects of vinegar on body weight and blood metabolites in healthy rats fed conventional diets and obese rats fed high-caloric diets. J. Med. Plants Res..

[35] Smith T.J. (2011). Green Tea Polyphenols in drug discovery—A success or failure?. Expert Opin. Drug Discov..

[36] Blasco T., Pérez-Burillo S., Balzerani F., Hinojosa-Nogueira D., Lerma-Aguilera A., Pastoriza S., Cendoya X., Rubio Á., Gosalbes M.J., Jiménez-Hernández N. (2021). An extended reconstruction of human gut microbiota metabolism of dietary compounds. Nat. Commun..

[37] Maron D.J., Lu G.P., Cai N.S., Wu Z.G., Li Y.H., Chen H., Zhu J.Q., Jin X.J., Wouters B.C., Zhao J. (2003). Cholesterol-lowering effect of a theaflavin-enriched green tea extract: A randomized controlled trial. Arch. Intern. Med..

[38] Shanafelt T.D., Call T.G., Zent C.S., LaPlant B., Bowen D.A., Roos M., Secreto C.R., Ghosh A.K., Kabat B.F., Lee M.J. (2009). Phase I trial of daily oral Polyphenon E in patients with asymptomatic Rai stage 0 to II chronic lymphocytic leukemia. J. Clin. Oncol..

[39] Stoicov C., Saffari R., Houghton J. (2009). Green tea inhibits Helicobacter growth in vivo and in vitro. Int J. Antimicrob. Agents.

[40] Baer D.J., Novotny J.A., Harris G.K., Stote K., Clevidence B., Rumpler W.V. (2011). Oolong tea does not improve glucose metabolism in non-diabetic adults. Eur. J. Clin. Nutr..

[41] Yuan X., Long Y., Ji Z., Gao J., Fu T., Yan M., Zhang L., Su H., Zhang W., Wen X. (2018). Green Tea Liquid Consumption Alters the Human Intestinal and Oral Microbiome. Mol. Nutr. Food Res..

[42] Bond T., Derbyshire E. (2019). Tea Compounds and the Gut Microbiome: Findings from Trials and Mechanistic Studies. Nutrients.

[43] Dajanta K., Janpum P., Leksing W. (2013). Antioxidant capacities, total phenolics and flavonoids in black and yellow soybeans fermented by Bacillus subtilis: A comparative study of Thai fermented soybeans (thua nao). Int. Food Res. J..

[44] Lee M., Hong G.-E., Zhang H., Yang C.-Y., Han K.-H., Mandal P.K., Lee C.-H. (2015). Production of the isoflavone aglycone and antioxidant activities in black soymilk using fermentation with Streptococcus thermophilus S10. Food Sci. Biotechnol..

[45] Zhao L., Zhang H., Hao T., Li S. (2015). In vitro antibacterial activities and mechanism of sugar fatty acid esters against five food-related bacteria. Food Chem..

